# Use of Emerging Technologies to Assess Differences in Outdoor Physical Activity in St. Louis, Missouri

**DOI:** 10.3389/fpubh.2014.00041

**Published:** 2014-05-23

**Authors:** Deepti Adlakha, Elizabeth L. Budd, Rebecca Gernes, Sonia Sequeira, James A. Hipp

**Affiliations:** ^1^Brown School, Washington University in St. Louis, St. Louis, MO, USA

**Keywords:** physical activity, parks, MapMyRun.com, socioeconomic status, web data feeds

## Abstract

**Introduction:** Abundant evidence shows that regular physical activity (PA) is an effective strategy for preventing obesity in people of diverse socioeconomic status (SES) and racial groups. The proportion of PA performed in parks and how this differs by proximate neighborhood SES has not been thoroughly investigated. The present project analyzes online public web data feeds to assess differences in outdoor PA by neighborhood SES in St. Louis, MO, USA.

**Methods:** First, running and walking routes submitted by users of the website MapMyRun.com were downloaded. The website enables participants to plan, map, record, and share their exercise routes and outdoor activities like runs, walks, and hikes in an online database. Next, the routes were visually illustrated using geographic information systems. Thereafter, using park data and 2010 Missouri census poverty data, the odds of running and walking routes traversing a low-SES neighborhood, and traversing a park in a low-SES neighborhood were examined in comparison to the odds of routes traversing higher-SES neighborhoods and higher-SES parks.

**Results:** Results show that a majority of running and walking routes occur in or at least traverse through a park. However, this finding does not hold when comparing low-SES neighborhoods to higher-SES neighborhoods in St. Louis. The odds of running in a park in a low-SES neighborhood were 54% lower than running in a park in a higher-SES neighborhood (OR = 0.46, CI = 0.17–1.23). The odds of walking in a park in a low-SES neighborhood were 17% lower than walking in a park in a higher-SES neighborhood (OR = 0.83, CI = 0.26–2.61).

**Conclusion:** The novel methods of this study include the use of inexpensive, unobtrusive, and publicly available web data feeds to examine PA in parks and differences by neighborhood SES. Emerging technologies like MapMyRun.com present significant advantages to enhance tracking of user-defined PA across large geographic and temporal settings.

## Introduction

Obesity has been recognized as a mounting public health challenge, increasing population risk of developing several chronic conditions (1, 2). In the United States (US), obesity is a leading cause of preventable death, second only to smoking (3). Persons of low socioeconomic status (SES) and a minority ethnic or racial background have a higher risk for obesity and related negative health consequences, compared to higher-SES and White populations (4, 5). Missouri has the 17th highest prevalence of adulthood obesity among US states (6). In the St. Louis, MO, US metro region, home to 2.8 million people, 29.8% of adults are obese (7).

Abundant evidence shows that regular physical activity (PA) is an effective strategy for reducing and preventing obesity in people of all SES and racial groups (8). The US National Park Services’ Healthy Parks Healthy People Strategic Action Plan 2011 describes health and well-being as an interrelated system linking human health to natural landscapes (9). Urban parks and green spaces provide opportunities for people to engage in various forms of PA (e.g., running, walking, bicycling) while connecting with the natural environment (10, 11). A recent study on PA and park use in five US cities found that up to 50% of weekly vigorous PA and 16% of weekly moderate PA was performed within parks (12). Studies have found that access to parks and green spaces is associated with increased quality of life and well-being (13, 14).

Evidence suggests disparities exist in park proximity, accessibility, and use (10, 15–17), as well as disparities in health outcomes in low-SES communities and among racial and ethnic minorities (18, 19). When controlling for mean neighborhood income, Suminski et al. (20) found that areas with higher percentages of racial/ethnic minorities had the least access to parks and fewest amenities within parks, in comparison with predominantly White neighborhoods. Although access to parks and green spaces is positively related to PA and negatively related to SES (10, 15, 16), the proportion of PA performed in parks and green spaces and how this differs by the proximate neighborhood SES has not been thoroughly investigated. Further, a majority of studies on PA and park use have used self-report surveys, observational audit tools, and activity logs wherein the resource and time-intensive nature of these traditional methods are key limitations (21–25). The present study (1) assesses the use of parks and green spaces for PA (bouts of running and walking) in a Midwestern US city (St. Louis, MO), and (2) examines if this park and green space use differs by the SES of the neighborhood surrounding the park. Understanding possible inequitable utilization of parks and green spaces is essential for public health and urban planning policies related to ameliorating health disparities.

## Materials and Methods

This study is the first of its kind to use unobtrusive, inexpensive, and publicly available web data feeds along with geographic information systems (GIS) methods to assess the use of parks and green spaces for PA and examine if park usage differs by neighborhood SES. This section provides details on: the study site; measurement of neighborhoods; sources of the data; data collection procedures; and data mapping procedures.

### Study site

The city of St. Louis, MO, US, is rich in terms of number of parks. According to the “2010 City Park Facts,” St. Louis has 9.6 acres of park per 1,000 residents, which is 50% more than Los Angeles and more than double both New York City and Chicago (26). St. Louis is far from rich in other ways, for example, data from the 2010 US Census show that St. Louis has a high poverty rate, with 27% of residents living below the federal poverty line (27). St. Louis also has significant adverse health indicators; the death rate is 14% higher than the rest of Missouri, 32% higher than the US, and heart disease mortality is 1.4 times the rate of the US (28). There are also stark differences by race and income between north and south St. Louis (see Figure 2 for SES distribution). A greater part of St. Louis considered in this study is classified as low-SES, and is primarily concentrated in the north.

Given lack of consensus on what defines a unique neighborhood (29), US census tracts were used as the primary definition of a neighborhood in this study. Census tracts in north St. Louis are 92–99% non-white (primarily African-American) with 38.6% of the population living below the federal poverty line (27). The personal wealth, economic opportunity, living conditions, and health outcomes are all significantly poorer in north St. Louis compared to south St. Louis (27). For example, African-American populations in St. Louis face higher rates of heart disease, cancer, cardiovascular disease, diabetes mortality, and have a life expectancy of 6.3 years fewer than White populations (28).

### Data sources

Physical activity was represented by bouts of walking, jogging, and running in this study. The website MapMyRun.com was the data source for running and walking routes. MapMyRun.com is a route mapping website that provides users worldwide with the ability to plan, map, record, and share their exercise routes, workouts, and outdoor activities like runs, walks, and hikes in an online database (30). Based on built-in geographic positioning system (GPS) technology, MapMyRun.com allows users to record activity using mobile applications compatible with electronic devices (e.g., smart phones), import data from third-party devices (e.g., wearable fitness tracking devices), or enter activity manually from a computer using an interactive map on its website. Users can specify the activity type (walking, running, hiking, dog walking, commuting, etc.) as well as the location. Details such as route start and end points, distance, elevation, points of interest, photographs, and other information of the activity can be recorded. In addition, users have access to a searchable database of over 80 million global routes, online training tools, nutrition tracking, fitness calculators, and event listings, with the ability to share their activities easily with others. For this analysis, we only used publicly available walking and running routes uploaded to MapMyRun.com in St. Louis during calendar year 2012.

Data on parks and green spaces were obtained from a variety of sources including the Environmental Systems Research Institute (31), and park departments in St. Louis City, St. Louis County, and additional municipalities within the jurisdiction of St. Louis County. Missouri census poverty data were obtained from the American Community Survey 2011 (5-year estimates) (27). Census tracts with 20% household poverty or higher were defined as low-SES per US Census Bureau guidelines (27).

### Data collection and analysis

Since 2006, 26,052 runs and walks have been posted on MapMyRun.com in St. Louis. During 2012, 80% of all PA bouts uploaded to MapMyRun.com in St. Louis were runs, with the remaining 20% of PA uploaded as walks. To capture seasonal variation, one route was downloaded per day across the 366 calendar days in 2012. A total of 71 walking routes (representing 20% of dates) and 287 running routes (representing 80% of dates) were systematically selected and downloaded for every day of 2012 from MapMyRun.com. Two dates are missing from walks and six from runs. These missing dates represent days in which the specified activity was not uploaded to MapMyRun.com in our study area.

Starting with the first day of each month, one running route was downloaded for each of four consecutive days (80%; e.g., one running route per day from January 1–4, 2012), followed by one walking route for the following consecutive day (e.g., one walking route from January 5, 2012). The process was repeated cyclically for all months in 2012, maintaining a ratio of four running routes to one walking route downloaded. The number of user uploaded routes for any given day varies on MapMyRun.com; a random number generator method (RANDSELECT in Microsoft Excel) was used to pick which route to download among several routes listed for a particular date.

The interactive map on MapMyRun.com permitted demarcation of study site boundaries within the larger St. Louis metropolitan area. The intersections of major interstate highways (Interstates 270 and 44, 270 and 70) were used to define the western edge of the study site. The Mississippi River, which is also the border between the neighboring states of Missouri and Illinois, defined the eastern edge of the study site. These boundaries captured all of St. Louis City and the majority of the population in St. Louis County (referred to collectively as St. Louis throughout this manuscript). The specific sample area was 903.30 km^2^ (348.77 miles^2^).

The built-in GPS technology on MapMyRun.com allowed for running and walking routes to be downloaded as GPS eXchange Format, or GPX files, which are a collection of points representing each route. These GPX files were imported into ArcGIS 10.0 (31) and layered over park data and Missouri SES census data (27). GIS spatial analysis was used to identify running and walking routes that occurred tangential to and/or within parks, and identify which of these routes occurred in parks located in low-SES census tracts. Buffers of 30 ft (9.14 m) were created around all parks to capture runs and walks occurring on sidewalks that border parks and to limit potential GPS signal strength error (32). For runs or walks that traversed one or more parks at some point during the route, the clipping tool in GIS was used to extract the length of each individual route segment that traversed a park. A sum total of the length of individual route segments that traversed a park was created for each user.

If a park crossed two or more census tract boundaries, the SES of the census tract with the highest percentage of park area was used to represent the SES surrounding the park. Logistic regression was used to examine the odds of running and walking routes traversing a low-SES neighborhood, and traversing a park in a low-SES neighborhood (dichotomous outcomes: yes/no) using Statistical Package for Social Sciences (SPSS) version 21 (33). Finally, a spatial autocorrelation was conducted in ArcGIS 10.0 (31) to assess the clustering of running and walking routes and parks.

## Results

**Figure 1:**
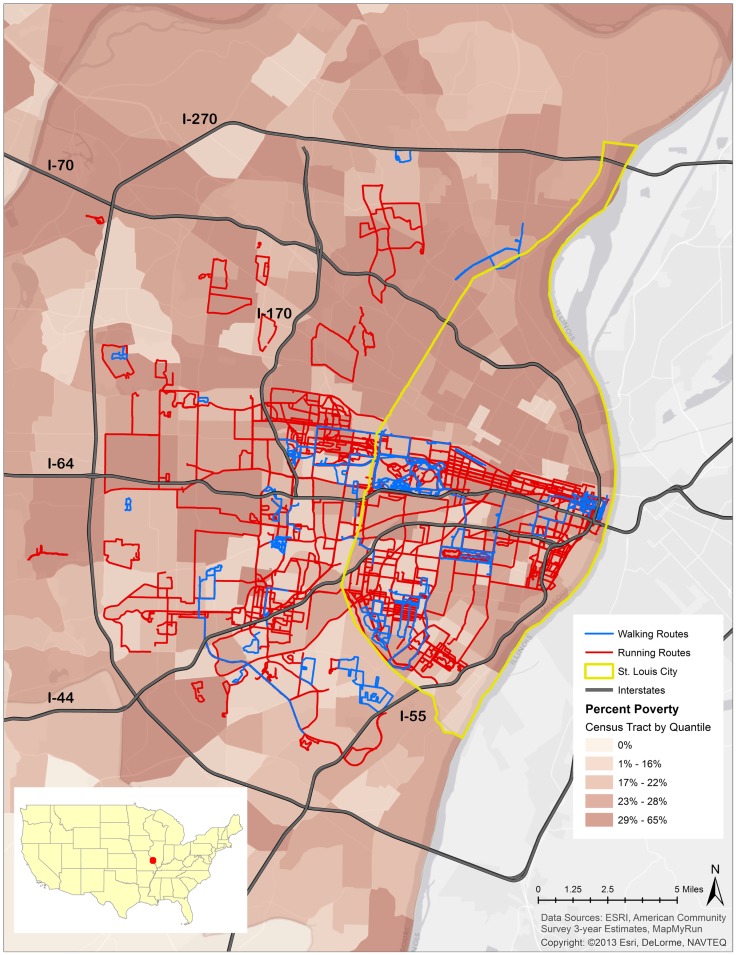
**Running routes, walking routes, and poverty rate in St. Louis, MO, USA**.

**Figure 2:**
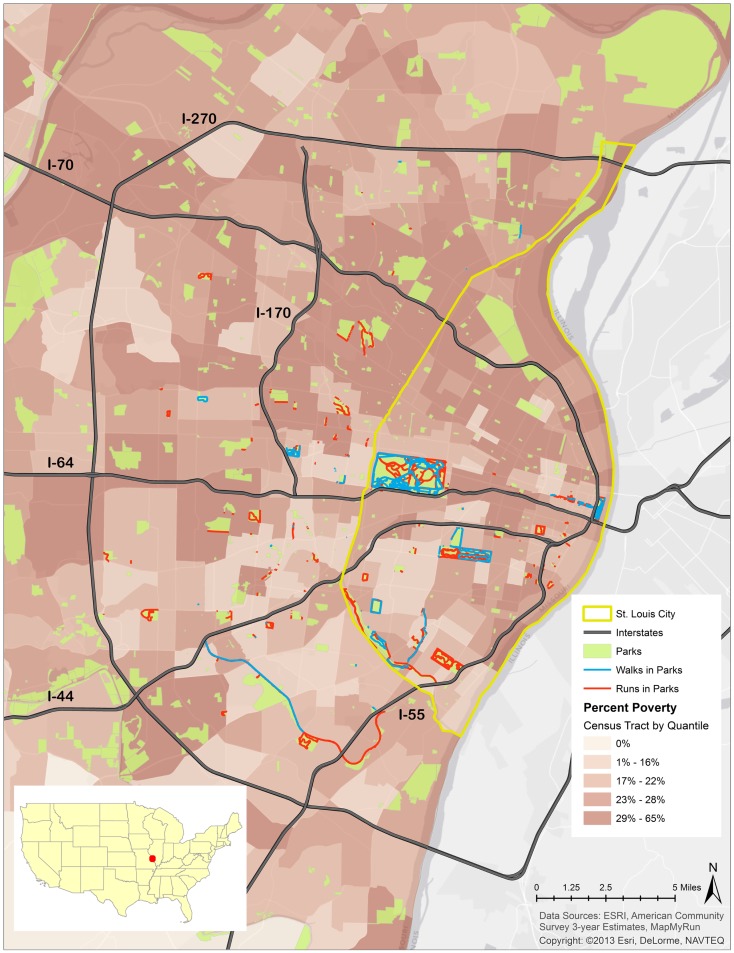
**Running and walking routes in parks and poverty rate in St. Louis, MO, USA**.

Results show that a large majority of running and walking routes were through or tangential to a park or green space. A total of 1,722.01 miles from 287 running routes and 236.84 miles from 71 walking routes appear in Figure 1 and Table 1. The average lengths of a run and walk in this sample were 6.00 and 3.33 miles respectively. 80.80% of runs traversed a park at some point during their run and 37.50% of these runs took place in parks located in low-SES neighborhoods (Table 1). Of the 71 walking routes, 70.40% traversed a park at some point during the walk and 27.43% of those walking routes took place in parks located in low-SES neighborhoods (Table 1). Figure 2 illustrates the availability of many parks across St. Louis, but shows fewer mapped running or walking routes in the northern half of the region that features more low-SES neighborhoods.

**Table 1:** **Use of parks in St. Louis, MO, USA for physical activity in 2012^a^**.

	Runs	Walks
*N*	287	71
Total distance (in miles)	1722.01	236.84
Distance (in miles) in parks	519.60	101.00
% in or tangential to parks	80.80	70.40
% in parks in low-SES neighborhoods	37.50	27.43

*^a^Running and walking routes downloaded from MapMyRun.com*.

The odds of running and walking routes traversing low-SES neighborhoods were significantly higher than the odds of running and walking routes reported in higher-SES neighborhoods (runs: OR = 2.64, CI = 1.58–4.39; walks: OR = 3.18, CI = 1.87–5.44) (Table 2). The odds of running in a park in a low-SES neighborhood were 54% lower than running in a park in a higher-SES neighborhood (OR = 0.46, CI = 0.17–1.23). The odds of walking reported in a park in a low-SES neighborhood were 17% lower than walking in a park in a higher-SES neighborhood (OR = 0.83, CI = 0.26–2.61).

**Table 2:** **Logistic regression: odds of running and walking in a low-SES neighborhood and park, compared to higher-SES neighborhoods in St. Louis, MO, USA in 2012**.

	*N*	OR	95% CI	*R*^2^ adj.
Runs in low-SES neighborhood	274	2.64***	1.58–4.39	0.07
Walks in low-SES neighborhood	274	3.18***	1.87–5.44	0.09
Runs traversing low-SES parks	173	0.46	0.17–1.23	0.02
Walks traversing low-SES parks	173	0.83	0.26–2.61	0.00

***p < 0.001.

The spatial autocorrelation indicated that running routes were significantly clustered (Moran’s Index = 1.22, *z*-score = 5.61, *p*-value = 0.00). Walking routes were not significantly clustered (Moran’s Index = 0.46, *z*-score = 1.38, *p*-value = 0.17), but trending toward significance. The distribution of parks (Moran’s Index = 0.00, *z*-score = 0.02, *p*-value = 0.98) was not significantly different than a random distribution.

## Discussion

This study showed that a majority of running and walking routes in St. Louis recorded by users of MapMyRun.com occur in, or at least traverse through, parks. However, this finding does not hold when comparing low-SES neighborhoods to higher-SES neighborhoods in St. Louis. By examining neighborhood-level socioeconomic disparities in the use of parks for PA, this study contributes important evidence to existing literature on PA and park use. The novel use of inexpensive, unobtrusive, and publicly available web data feeds to assess the use of public parks and green spaces for outdoor PA is a main strength of this study.

Community health research has shown that parks have significant roles in supporting up to 50% of the moderate-to-vigorous PA of the local population (8, 12). Findings from this study show a higher percentage of PA in the form of runs and walks occurring in parks, with approximately 80% of running and 70% of walking routes traversing a park at some point. While previous studies refer to total PA (12), this study considered only bouts of walking and running to represent PA. The higher percentage of runs and walks occurring in parks indicate that other types of PA (e.g., hockey, swimming, and weight training) may be occurring in parks at much lower rates than walking and running. The spatial clustering of running routes indicates significant differences from what would be expected by chance, suggesting that specific areas in St. Louis (parks, as evident from Figure 2) are more likely to be sought as venues for running. The insignificant spatial clustering of walking routes can be partially attributed to fewer numbers and miles of walking routes considered in this analysis. Overall, findings from this study support and extend the existing knowledge base on the role of parks and green spaces as venues for outdoor PA, particularly running and walking.

Previous studies have shown that low-SES neighborhoods are less likely to have available facilities and locations to facilitate PA, such as parks and green spaces (15, 19). Contrary to the literature, the present results indicate increased odds of running and walking in low-SES St. Louis neighborhoods compared to higher-SES St. Louis neighborhoods (Table 1). The high percentage of low-SES census tracts in the St. Louis metropolitan area in this analysis may be a possible explanation for the higher odds of running and walking bouts occurring in low-SES areas in St. Louis (Table 2).

The lower odds of running and walking in parks in low-SES neighborhoods compared to parks in higher-SES neighborhoods further corroborates several health and environmental disparities between north and south St. Louis. Previous studies in other metropolitan cities have indicated that low-SES neighborhoods have access to the fewest acres of parks and green spaces (20, 34). On the contrary, this study illustrates that low-SES neighborhoods in north St. Louis have proximate access to several parks and green spaces, equal to higher-SES south St. Louis neighborhoods, yet parks and green spaces in north St. Louis remain underutilized.

Our analysis indicates a random, non-clustered distribution of parks across the St. Louis region. However, many of the parks, particularly those in north St. Louis, remain underutilized for running and walking (Figure 2). Perceived constraints to park use may be a possible explanation for the underutilization of parks and green spaces as venues for engagement in PA in north St. Louis. Perceived crime is a common constraint identified by individuals who live near parks but do not use them (17, 34, 35). A qualitative study on perceived constraints to park use in two north St. Louis communities, both within close proximity of a public park, highlighted several issues related to maintenance, safety, and limited amenities that constrained park use and subsequent healthy behaviors (17). Studies have suggested that proximity to parks and green spaces is predictive of nearby residents’ PA within the park (11). Decreasing barriers to using parks in low-SES areas like north St. Louis can enhance park-based PA among neighborhood residents. Increased PA in low-SES areas can contribute to reducing disparate rates of obesity and related chronic diseases and improving population health outcomes in these areas.

Increasingly, the demand for infrastructure to accommodate growing populations in many cities and towns has been achieved through the modification or demolition of parks and green spaces (36, 37). Approximately 3,500 acres of parks in St. Louis were recently threatened with closures due to budget cuts (38, 39). Findings from this study show the preference of parks and green spaces as venues for walking and running by a majority of people in this sample, making a compelling case for improved budgetary support toward park conservancy efforts in St. Louis.

### Strengths and limitations

The unobtrusive and objective nature of data obtained from MapMyRun.com is an important strength of this study that has the potential to advance PA measurement by placing minimum burden on the sample population. MapMyRun mobile applications can be downloaded free of cost and are supported by a variety of electronic devices and platforms (e.g., Android, Apple, Windows, Blackberry platforms), freely permitting large-scale data collection across widespread geographic areas. The extensive and objective nature of this data has potential for longitudinal studies in future PA research. Additionally, information on routes such as distance, speed, elevation, origin, and destination can allow researchers to conduct further detailed investigation on substantially larger samples of PA behavior across extensive geographic locations.

Existing public health literature on PA in parks focuses on park proximity and use, but little is known about the absolute amount or types of PA they facilitate and demographic and SES characteristics of the populations they serve. An exception to this are studies using direct observation instruments like the System for Observing Physical Activity and Recreation in Communities (SOPARC) (22). Instruments like SOPARC have been able to provide objective, contextually rich information on PA in parks and other open environments, but these data are static since parks are divided into predetermined target areas and then studied by trained observers. Other limitations of direct observation instruments are the time-intensive nature and costs involved in data collection (25). In contrast, data from MapMyRun.com provides a cheaper alternative for precise tracking of PA across larger spatial and temporal settings.

Despite the above advantages, this approach presents several limitations. Data from websites like MapMyRun.com are limited to people with access to some form of GPS technology, those that select to map their running and walking routes, and users who choose to make them publicly available online. Populations who use such technology to monitor their PA may be comparatively more health conscious than the general population.

While a key strength of this study is that it addresses the increasing use of technology to map PA behaviors, it is limited in that certain populations may be more likely than others to use websites like MapMyRun.com. The use of emerging technologies is known to vary by SES, gender, age, ethnicity, and other factors (40). Overall, the use of smartphones and other emerging technologies like MapMyRun.com to monitor and evaluate PA behaviors has been steadily increasing. Telecommunication data shows that 56.80% of US mobile phone users in 2013 were smartphone users, projected to increase by another 15% by 2015 (40).

Several demographic groups have high levels of smartphone adoption. Among low-SES populations, smartphone ownership rates between the ages 18 and 29 are equal to the national average. However, among non-White populations, smartphone usage is less than the national average; only 44% of African-Americans and Latinos are smartphone users (41). Low-SES ethnic minority populations may have limited access to resources and awareness of emerging technologies like MapMyRun.com and other health-related mobile applications to map their PA behaviors. This may be another reason for fewer mapped running and walking routes in north St. Louis. There is no publically available information on SES characteristics of users of MapMyRun.com which limits generalizability of findings.

Pertinent to this study, the occurrence of walking or running routes within or tangential to a park, route origin, and destination points, and the choice of the parks themselves cannot be linked with residential proximity and park use. Users could be relying on a form of motorized transport (e.g., car) to travel to a park located in a census tract different from the one they reside or work in, and then complete a walk or run in the selected park, recording its details using GPS technology. Making assumptions about individual behaviors based on aggregate data from MapMyRun.com is vulnerable to the phenomenon of ecological fallacy. Although this study indicates higher odds of walking and running in low-SES areas compared to higher-SES areas, inferences about the SES characteristics of the population cannot be made; residents from higher-SES census tracts may be running and walking in low-SES areas, thus limiting the ability to generalize these findings. The exclusion of any other PA types that may be occurring in parks (e.g., sports like soccer, golf, tennis, etc.) constrains the estimation of total PA in this study. The lack of data on availability and quality of sidewalks is another limitation of this study since it did not permit comparisons related to sidewalk access, sidewalk connectivity to parks, and subsequent park usage between low-SES and high-SES neighborhoods.

Methodological constraints in the process of downloading routes from MapMyRun.com may also impact generalizability of findings. There is no way of filtering the routes posted on MapMyRun.com by specific users. However, as of April 2014, there were 3,349 unique MapMyRun users who have listed their home as St. Louis, MO, USA. That stated, we are unable to identify the average number of routes created per user which impairs the ability to make generalizable statements.

Accuracy of data and signal strength in the use of GPS technology are known to be a weakness, albeit improving. Another major disadvantage in the use of technologies like MapMyRun.com to track PA is its potential to influence behavior among users. For example, users may alter route distance, speed, location, etc., when using GPS technology to track their PA behavior. Results from this study are therefore limited to users of MapMyRun.com, their PA patterns and route selections, and cannot be generalized to the larger population. Despite limited generalizability, data from MapMyRun.com have the potential to reveal distinct patterns of park use and non-park use. Further research is needed to identify underlying reasons for these patterns.

The use of publicly available web data feeds from MapMyRun.com also raises key ethical and privacy considerations. While user names and addresses are not public on MapMyRun.com, mapped GPS data can reveal underlying patterns in route characteristics such as route origin, time and frequency of occurrence, distance, speed, etc. Researchers using emerging technologies should be cognizant of this and work with ethicists and Institutional Review Boards to ensure privacy and confidentiality of users.

Overall, the unobtrusive nature of data from MapMyRun.com offers several opportunities to provide an unbiased sample of PA patterns in outdoor environments. Next steps include validation of data collected from MapMyRun.com, a detailed examination of park quality and features, and the identification of specific built environment attributes in low-SES neighborhoods that are the most crucial in influencing PA patterns.

## Conclusion

This study is novel in its use of an emerging technology like MapMyRun.com to track user-defined PA routes in parks and differences in occurrence of these routes by neighborhood SES. Future research could assess additional factors (e.g., quality of parks, neighborhood infrastructure) and their relationship to PA across larger geographical areas and over extended periods of time. A more nuanced understanding of PA in parks is needed for better attribute, policy, and programmatic solutions to increase use of parks for PA, especially those located in low-SES neighborhoods where health disparities are greatest.

## Conflict of Interest Statement

The authors declare that the research was conducted in the absence of any commercial or financial relationships that could be construed as a potential conflict of interest.
