# Corrigendum: Use of Emerging Technologies to Assess Differences in Outdoor Physical Activity in St. Louis, Missouri

**DOI:** 10.3389/fpubh.2015.00041

**Published:** 2015-03-16

**Authors:** Deepti Adlakha, Elizabeth L. Budd, Rebecca Gernes, Sonia Sequeira, James A. Hipp

**Affiliations:** ^1^Brown School, Washington University in St. Louis, St. Louis, MO, USA

**Keywords:** physical activity, parks, MapMyRun.com, socioeconomic status, web data feeds

Results and figures of the article by Adlakha et al. (2014) contained minor errors, which we hereby rectify.

Results show that a large majority of running and walking routes were through or tangential to a park or green space. A total of 1,722.01 miles from 287 running routes and 236.84 miles from 71 walking routes appear in Figure 1 and Table 1. The average lengths of a run and walk in this sample were 6.00 and 3.33 miles, respectively. Of all the parks in the study area, 70% were located in low-income neighborhoods. Of the 287 running routes, 80.80% traversed a park at some point during the run and 6.97% of these runs took place in parks located in low-SES neighborhoods. Of the 71 walking routes, 70.40% traversed a park at some point during the walk and 15.50% of walking routes occurred in parks located in low-SES neighborhoods. Figure 2 illustrates the availability of many parks across St. Louis, but shows fewer mapped running or walking routes in the northern half of the region that features more low-SES neighborhoods.

**Figure 1:**
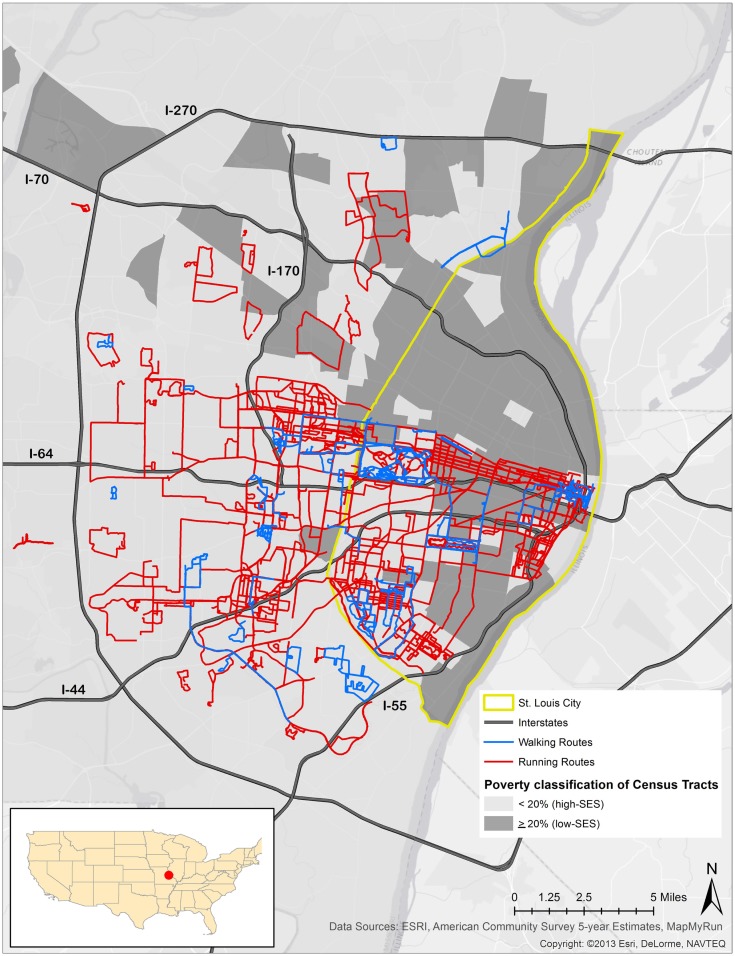
**Running routes, walking routes, and poverty rate in St. Louis, MO, USA**.

**Table 1:** **Use of parks in St. Louis, MO for physical activity in 2012^a^**.

	Runs	Walks
*N*	287	71
Total distance (in miles)	1722.01	236.84
Distance (in miles) in parks	519.60	101.00
% in or tangential to parks	80.80	70.40
% in parks in low-SES neighborhoods	6.97	15.50

*^a^Running and walking routes downloaded from MapMyRun.com*.

**Figure 2:**
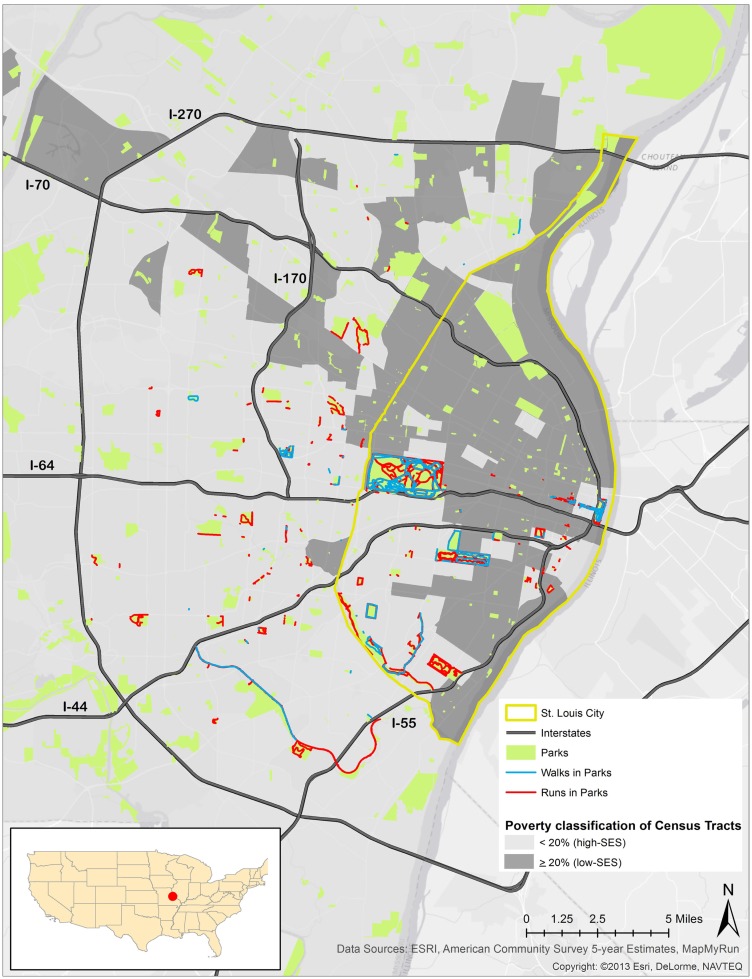
**Running and walking routes in parks and poverty rate in St. Louis, MO, USA**.

The odds of reported running and walking routes traversing low-SES neighborhoods were significantly lower than the odds of running and walking routes reported in higher-SES neighborhoods (runs: OR = 0.36, CI = 0.21–0.62; walks: OR = 0.41, CI = 0.23–0.73) (Table 2). The odds of running in a park in a low-SES neighborhood were 52% lower than running in a park in a higher-SES neighborhood (OR = 0.48, CI = 0.29–0.79). The odds of walking reported in a park in a low-SES neighborhood were 64% lower than walking in a park in a higher-SES neighborhood (OR = 0.36, CI = 0.16–0.82).

**Table 2:** **Logistic regression: odds of running and walking in a low-SES neighborhood (*N* = 238) and park (*N* = 511), compared to higher-SES neighborhoods**.

	*N*	OR	95% CI	*R*^2^ adj.
Runs in low-SES neighborhood	238	0.36***	0.21–0.62	0.06
Walks in low-SES neighborhood	238	0.41**	0.23–0.73	0.04
Runs traversing low-SES parks	511	0.48**	0.29–0.79	0.02
Walks traversing low-SES parks	511	0.36*	0.16–0.82	0.01

*p < 0.05.

**p < 0.01.

***p < 0.001.

Revised results indicate decreased odds of reported running and walking in low-SES St. Louis neighborhoods compared to higher-SES St. Louis neighborhoods (Table 1). This finding is consistent with the disparate rates of PA in low versus higher-SES areas (1, 2). Overall, the lower odds of reported running and walking in low-SES neighborhoods and parks located in these low-SES neighborhoods compared to higher-SES neighborhoods and parks corroborates several health and environmental disparities between north and south St. Louis.

## Conflict of Interest Statement

The authors declare that the research was conducted in the absence of any commercial or financial relationships that could be construed as a potential conflict of interest.
